# Regression and Classification With Spline-Based Separable Expansions

**DOI:** 10.3389/fdata.2022.688496

**Published:** 2022-02-11

**Authors:** Nithin Govindarajan, Nico Vervliet, Lieven De Lathauwer

**Affiliations:** ^1^Center for Dynamical Systems, Signal Processing and Data Analytics, Department of Electrical Engineering (ESAT), KU Leuven, Leuven, Belgium; ^2^Group Science, Engineering and Technology, KU Leuven Kulak, Kortrijk, Belgium

**Keywords:** B-splines, tensor decompositions, canonical polyadic decomposition, supervised learning, gauss-newton, classification, regression

## Abstract

We introduce a supervised learning framework for target functions that are well approximated by a sum of (few) separable terms. The framework proposes to approximate each component function by a B-spline, resulting in an approximant where the underlying coefficient tensor of the tensor product expansion has a low-rank polyadic decomposition parametrization. By exploiting the multilinear structure, as well as the sparsity pattern of the compactly supported B-spline basis terms, we demonstrate how such an approximant is well-suited for regression and classification tasks by using the Gauss–Newton algorithm to train the parameters. Various numerical examples are provided analyzing the effectiveness of the approach.

## 1. Introduction

Approximating multivariate functions in high dimensions quickly becomes infeasible due to the curse of dimensionality. Negative results in the literature (DeVore et al., 1989; Yarotsky, 2017) reveal that to approximate a generic *n*-times differentiable function in *D* variables within ϵ-tolerance (measured in the uniform norm), one typically would require *M* ≳ ϵ^−*D*/*n*^ parameters. Fortunately, in machine learning practice, many target functions are of inherently low complexity if examined through the right lens. That is, by selecting a suitable architecture for the approximant such that it resembles the underlying structure of the true function, the curse of dimensionality can typically be avoided. For instance, a common architecture, adapted particularly by deep neural networks (Schmidhuber, 2015), is to express the approximant as a sequence of compositions of simpler functions. The success of such kind of networks in supervised learning tasks has been profound and can be largely attributed to the fact that many phenomena in nature are the result of a sequence of simpler operations; see (Mhaskar and Poggio, 2016).

In this paper, we study another commonly occuring structure in which the target function *f*(*x*) essentially has low rank and can be expressed as a sum of few separable terms, i.e.,


(1) $$\begin{array}{l} f\left(\text{x}\right)=\overset{R}{\underset{r=1}{\sum }}\left(\overset{D}{\underset{d=1}{\prod }}\phi_{r}^{\left(d\right)}\left(x_{d}\right)\right)=:〚\Phi^{\left(1\right)}\left(x_{1}\right),\ldots ,\Phi^{\left(D\right)}\left(x_{D}\right)〛, \end{array}$$

where $\Phi^{\left(d\right)}\left(x_{d}\right)\;=:\;\left[\begin{array}{ccc} \phi_{1}^{\left(d\right)}\left(x_{d}\right) & \cdots & \phi_{R}^{\left(d\right)}\left(x_{d}\right) \end{array}\right]$ and $\text{x}=\left[\begin{array}{ccc} x_{1} & \cdots & x_{D} \end{array}\right]$. Sums of separable functions describe continuous, infinite-dimensional analogs of (canonical) polyadic decompositions (CPD) of higher-order tensors (Kolda and Bader, 2009; Cichocki et al., 2015; Sidiropoulos et al., 2017), and a direct discretization of the univariate component functions $\phi_{r}^{\left(d\right)}\left(x_{d}\right)$ results in an approximant that takes the form of a tensor product expansion, where the underlying coefficient tensor has a low-rank polyadic decomposition parameterization. Low-rank approximations are a key ingredient to the success of tensor-based scientific computing (Hackbusch, 2012; Grasedyck et al., 2013; Khoromskij, 2018), and they provide a means to capture low complexity patterns in higher-dimensional datasets.

We present our initial findings on how one can effectively approximate functions of the type (Equation 1) in the context of supervised learning, appearing either in the form of regression or classification tasks. As opposed to earlier work on this topic (Beylkin et al., 2009; Garcke, 2010; Kargas and Sidiropoulos, 2021), a key feature in our framework is the choice to use B-splines or, equivalently, piecewise polynomials, as a means to discretize the individual component functions. B-splines have been a fundamental tool in computational geometry and numerical analysis for many years (De Boor, 1978), however, their explicit usage in pure machine learning applications has been fairly limited, despite the close connections between deep ReLU networks and nonuniform linear splines with adaptive knots (Unser, 2019). B-splines satisfy, in comparison to approximation by pure polynomials, some favorable properties as they are compactly supported—B-splines are nonzero only on a small interval—and allow for more adaptive local approximation of nonlinearities in a function. Furthermore, they are less prone to the unwanted high-oscillatory behavior that may occur in interpolatory fits of function data using high-degree polynomials.

We present an optimization framework to train the model parameters of the proposed approximant given data samples of a specific regression or classification problem. By introducing the low-rank structure, the problem can be viewed as a special case of a linear system with a CPD constrained solution (Boussé et al., 2018) or the computation of a CPD of an incomplete tensor with linearly constrained factor matrices (Vervliet et al., 2017). By exploiting the resulting multilinear structure of the quadratic objective function in a similar way as in Boussé et al. (2018) and Vervliet et al. (2017), we show that the training of the model parameters in the regression problem can be handled effectively with the Gauss–Newton (GN) algorithm with dog-leg trust region (Nocedal and Wright, 2006). In comparison to prior work which used alternating least squares (ALS) scheme (Beylkin et al., 2009; Garcke, 2010) as means to train the model parameters, the GN algorithm is known to exhibit superior convergence properties (see e.g., Sorber et al., 2013; Vervliet and De Lathauwer, 2019). Building on the results for the GN-based computation of a CPD using alternative cost functions (Vandecappelle et al., 2021), we show that our algorithm can be altered to accommodate logistic cost functions which are more suitable for classification problems.

The use of tensor decompositions in machine learning problems has been explored in various directions in the past. In Lebedev et al. (2015) and Jaderberg et al. (2014) tensor decompositions were used to accelerate convolutional layers of a neural network. Furthermore, the concept of using tensor decompositions is closely related to sum-product networks (Delalleau and Bengio, 2011; Poon and Domingos, 2011; Gens and Domingos, 2012; Gens and Pedro, 2013). Apart from the former work on sums of separable functions (Beylkin et al., 2009; Garcke, 2010; Kargas and Sidiropoulos, 2021), the utility of other types of tensor decompositions have also been studied in the literature. In Hendrikx et al. (2019), symmetric tensor decompositions were used to exploit structure in multivariate polynomial functions. In Grelier et al. (2018), Liu et al. (2019), and Hou and Chaib-Draa (2015), (hierachical)-Tucker decompositions were considered in machine learning contexts, whereas (Oseledets, 2013; Novikov et al., 2016; Chen et al., 2017) looked into tensor train decompositions instead. More general tensor networks were also examined in Reyes and Stoudenmire (2021) and Stoudenmire and Schwab (2016). Adaptive methods to learn tensor networks from data has also been studied in Hashemizadeh et al. (2020). Finally, Karagoz and Batselier (2020) considered using B-splines in combination with tensor networks for applications in system identification. However, none of the prior works have considered using B-splines in the context of machine learning applications.

The paper is organized as follows. First, in Section 2, we cover some basic mathematical preliminaries on B-splines, low-rank separable expansions, and tensors. Subsequently, the regression and classification frameworks are introduced in Sections 3 and 4, respectively. Herein, we cover also some numerical examples illustrating the utility of the proposed learning framework. The conclusions are provided in Section 5.

### Notation

The following notation is adopted throughout this paper. The symbol ℝ is reserved to denote the reals. *C*([0, 1]^*D*^) is used to denote the space of continuous function on the unit hypercube [0, 1]^*D*^. Scalars, (column) vectors, matrices and tensors are denoted by lowercase, bold lowercase, bold uppercase and calligraphic characters, respectively; e.g., *s* ∈ ℝ, **v** ∈ ℝ^*I*^, **M** ∈ ℝ^*I*×*J*^ and $T\in {\mathbb{R}}^{I_{1}\times I_{2}\times \ldots \times I_{D}}$. ${\text{1}}_{N}\in {\mathbb{R}}^{N}$ denotes the vector with all entries set to one. The symbols ⊗ and ⊗ denote the outer product and Kronecker product, respectively. The symbol *, on the other hand, denotes the Hadamard (or element-wise) product. The mode-*d* tensor-matrix product ·_*d*_ between a tensor $T\in {\mathbb{R}}^{I_{1}\times I_{2}\times \ldots \times I_{D}}$ and a matrix $\text{M}\in {\mathbb{R}}^{J\times I_{d}}$ has the following elementwise definition for $S=T\cdot_{d}\text{M}\in {\mathbb{R}}^{I_{1}\times \ldots \times I_{d-1}\times J\times I_{d+1}\times \ldots I_{D}}$:


$$\begin{array}{l} s_{i_{1},\ldots ,i_{d-1},j,i_{d+1},\ldots ,i_{D}}=\overset{I_{D}}{\underset{i_{d}=1}{\sum }}t_{i_{1},\ldots ,i_{d-1},i_{d},i_{d+1},\ldots ,i_{D}}m_{j,i_{d}}. \end{array}$$

A polyadic decomposition can be written compactly as


$$\begin{array}{l} 〚\Gamma^{\left(1\right)},\ldots ,\Gamma^{\left(D\right)}〛: =\overset{R}{\underset{r=1}{\sum }}\gamma_{r}^{\left(1\right)}⊗\cdots ⊗\gamma_{r}^{\left(D\right)}, \end{array}$$

where factor matrix $\Gamma^{\left(d\right)}=\left[\begin{array}{ccc} \gamma_{1}^{\left(d\right)} & \cdots & \gamma_{R}^{\left(d\right)} \end{array}\right]$ collects factor vectors $\gamma_{r}^{\left(d\right)}$ as its columns. The bracket notation is also used to compactly express sums of separable functions, i.e.,


$$\begin{array}{l} 〚\Phi^{\left(1\right)}\left(x_{1}\right),\ldots ,\Phi^{\left(D\right)}\left(x_{D}\right)〛:=\overset{R}{\underset{r=1}{\sum }}\left(\overset{D}{\underset{d=1}{\prod }}\phi_{r}^{\left(d\right)}\left(x_{d}\right)\right), \end{array}$$

where $\Phi^{\left(d\right)}\left(x_{d}\right)=\left[\begin{array}{ccc} \phi_{1}^{\left(d\right)}\left(x_{d}\right) & \cdots & \phi_{R}^{\left(d\right)}\left(x_{d}\right) \end{array}\right]$.

## 2. Preliminaries: B-splines, Low Rank Separable Expansions and Tensors

In this section, we cover the basic mathematical prelimaries for our proposed regression and classification frameworks which are discussed in Sections 3 and 4, respectively.

### 2.1. B-splines

A spline is a piecewise polynomial function stitched together in a way such that it maintains a certain degree of smoothness over the approximation interval. A convenient way of expressing splines is through B-splines. A B-spline of order *N* on the interval [*a, b*] is constructed as follows. Let 
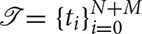
 denote the set of knots which satisfy the order relation


$$\begin{array}{l} a=t_{0}=\ldots =t_{N-1}\leq t_{N}\leq t_{N+1}\leq \ldots \leq t_{M+1}=\ldots \\ \;=t_{M+N}=b. \end{array}$$

The B-spline basis terms ${\left\{B_{m,N}\right\}}_{m=0}^{M}$ are given by the recursion formula


(2) $$\begin{array}{l} B_{m,N}\left(x\right): =\frac{x-t_{m}}{t_{m+N}-t_{m}}B_{m,N-1}\left(x\right)+\frac{t_{m+N+1}-x}{t_{m+N+1}-t_{m+1}}B_{m+1,N-1}\left(x\right), \end{array}$$

where


$$B_{m,0}\left(x\right): =\{\begin{array}{cc} 1 & x\in \left[t_{m},t_{m+1}\right)\\ 0 & \text{otherwise} \end{array}.$$

**Figure 1:**
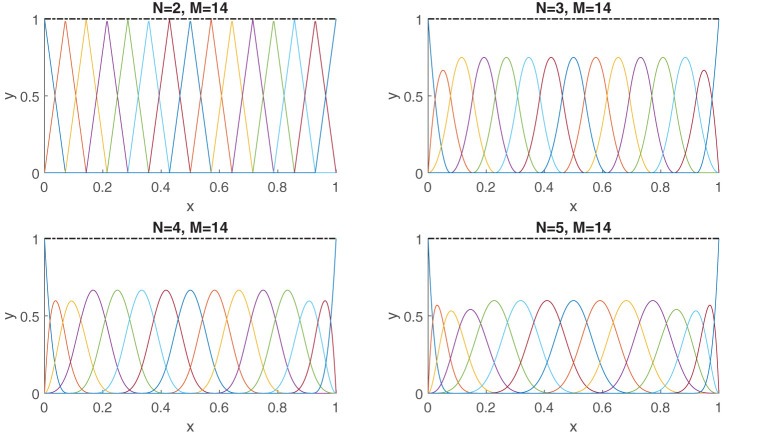
The B-spline basis terms (Equation 2) are compactly supported. Shown are the basis terms for orders *N* = 2, 3, 4, 5. The knots 
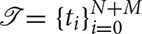
 are distributed uniformly: *t*_*i*_ = 0 for *i* = 0, …, *N* − 1, *t*_*i*_ = (*i* − *N* + 1)/(*M* − *N* + 2) for *i* = *N*, …, *M*, and *t*_*i*_ = 1 for *i* = *M* + 1, …, *N*. Shown also in black is the sum of all the B-spline basis terms, i.e., $\sum_{m=0}^{M}B_{m,N}\left(x\right)=1$.

In Figure 1, the B-spline basis terms are graphically depicted for various orders and knot configurations. The B-spline basis terms satisfy the interesting property of being compactly supported. In particular, *B*_*m,N*_ can only attain nonzero values on the interval [*t*_*m*_, *t*_*m*+*N*+1_), i.e.,


(3) $$\begin{array}{l} B_{m,N}\left(x\right)=0,\;x\in \left(-\infty ,t_{m}\right)\cup \left[t_{m+N+1},\infty \right). \end{array}$$

To put this into perspective, a polynomial basis can never satisfy such a property: if a polynomial function is zero on a nonempty interval, then it must be zero everywhere. A general B-spline *S*(*x*) of order *N* is formed by taking weighted linear combinations of the basis elements (Equation 2). We have the expression:


$$\begin{array}{l} S\left(x\right)=\overset{M}{\underset{m=0}{\sum }}c_{m}B_{m,N}\left(x\right), \end{array}$$

which we denote more concisely in the vectorized notation



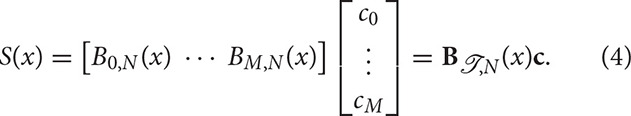



For the sake of readability, we omit the subscripts 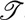, *N* in 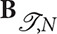 in the remainder of the text, i.e., **B**(*x*) = 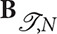
(*x*). However, one must keep in mind that a spline always implicitly involves a set of knots 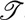 and an order *N*.

It is well-known that Equation (4) is a universal approximator and has the ability to approximate any continuous function with arbitrary accuracy (De Boor, 1978). This fact should come as no surprise, since (Equation 4) describes a piecewise degree *N* − 1 polynomial, and polynomials themselves are universal approximators as a consequence of the Weierstrass theorem. However, there is a fundamental feature of splines which allow functions to be approximated in a way that pure polynomials cannot: while the approximation power of pure polynomials can only be increased by raising the degree, in the case of splines, one has additional flexibility to raise the total number of polynomial pieces used on the approximation interval. This increase is effectively realized by increasing the knot density.

There are several advantages of being able to control the amount of polynomial pieces. First, it allows for local approximation of regions over which the function experiences dramatic change, since changes to the coefficients in Equation (4) affect the function only locally. Second, by keeping the degree low and increasing the number of knots, one can avoid some of the numerical issues faced by high-degree polynomials. A major drawback of high-degree polynomials is the possible appearance of large (non-physical) oscillations in an interpolatory fit of a function which increase in magnitude as the polynomial degree increases. This undesirable property is commonly referred to as Runge's phenomenon in the approximation theory literature (see e.g., Trefethen, 2019) for a more detailed exposition on the subject. In the context of machine-learning, large oscillations between the sample points can have an adverse effect on the generalization error. Henceforth, any use of high-degree polynomials in such contexts must keep Runge's effects at check, i.e., the method of approximation becomes of paramount approximation. This can be achieved in various ways; e.g., by overparameterizing the polynomial and adding a derivative penalty to the polynomial fit (Chandrasekaran et al., 2013; Rajagopal, 2019), or by using structured interpolation points such as Chebyshev nodes if one has the freedom to pick the function samples freely (Trefethen, 2019). Another alternative is to switch to splines, in which case, the issue is completely bypassed altogether if one keeps the order of the spline low. Third, resolving local phenomena such as kinks, discontinuities, and sharp transitions is difficult using polynomials due to their global nature. Splines, however, can capture local phenomena more easily thanks to their compact support, particularly if the knots are chosen to align with the boundaries of such features.

The performance of a given B-spline for a regression task is dependent on the chosen set of knots and order of the spline. In practice, one typically wants to optimize the number of knots as well as their respective positions in order to avoid under or overfitting of the data. Typically, the idea is to increase the knot frequency at data-dense regions where the underlying function tends to exhibit a high degree of variation. The problem of optimal knot allocation is, however, highly non-trivial and the literature has suggested various approaches to do adaptive knot selection (see Zhou and Shen, 2001) and the references therein. Fortunately, in most cases, decent performance can already be achieved by distributing the knots evenly with respect to the provided data (De Boor, 1978).

### 2.2. Multivariate Splines and Tensors

There are several ways to extend B-splines to the multivariate case. Instead of forming piecewise polynomials on a set of intervals, one constructs a smooth function in a higher-dimensional space by stitching together piecewise polynomials on a collection of polygonal domains. One well-studied framework is to define splines on so-called triangulations which partitions a domain into simplices (Lai and Schumaker, 2007).

A more basic approach, which is more amenable to our pursuit of low-rank approximations, is to take Cartesian tensor products of univariate B-splines. This effectively results in a construction where polynomials are defined piecewise on box-shaped domains. To define a tensor product B-spline on a feature space [*a*_1_, *b*_1_] × … × [*a*_*D*_, *b*_*D*_], a set of knots 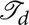 for each variable *x*_*d*_, *d* = 1, …, *D*, is defined first. A multivariate function can then be approximated by


$$\begin{array}{l} S\left(\text{x}\right)=\overset{M_{1}}{\underset{m_{1}=0}{\sum }}\overset{M_{2}}{\underset{m_{2}=0}{\sum }}\cdots \overset{M_{D}}{\underset{m_{D}=0}{\sum }}c_{m_{1}m_{2}\cdots m_{D}}\overset{D}{\underset{d=1}{\prod }}B_{m_{d},N_{d}}^{\left(d\right)}\left(x_{d}\right). \end{array}$$

The above expansion can be expressed more concisely by using tensor notation. With the help of mode-*d* tensor-matrix products, we write


(5) $$\begin{array}{l} S\left(\text{x}\right)=C\cdot_{1}{\text{B}}^{\left(1\right)}\left(x_{1}\right)\cdot_{2}{\text{B}}^{\left(2\right)}\left(x_{2}\right)\cdots \cdot_{D}{\text{B}}^{\left(D\right)}\left(x_{D}\right), \end{array}$$

where $C\in {\mathbb{R}}^{\left(M_{1}+1\right)\times \ldots \times \left(M_{D}+1\right)}$ denotes the *D*th order coefficient tensor, and ${\text{B}}^{\left(d\right)}\left(x_{d}\right)=\left[\begin{array}{ccc} B_{1,N_{d}}^{\left(d\right)}\left(x_{d}\right) & \cdots & B_{M_{d},N_{d}}^{\left(d\right)}\left(x_{d}\right) \end{array}\right]$ for some knot set 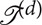, *d* = 1, …, *D*. Similar to how (Equation 4) is an universal approximator of any continous function on an interval, Equation (5) describes an universal approximator for any multivariate continuous function on the feature space [*a*_1_, *b*_1_] × ⋯ × [*a*_*D*_, *b*_*D*_]. Likewise, as a consequence of Equation (3), the basis elements of Equation (5) are also compactly supported.

### 2.3. Low-Rank Separable Expansions and CPDs

The number of entries in the coefficient tensor $C\in {\mathbb{R}}^{\left(M_{1}+1\right)\times \cdots \times \left(M_{D}+1\right)}$ equals $\prod_{d=1}^{D}\left(M_{d}+1\right)$ and grows exponentially with the dimension *D* of the function domain. This exponential blow-up of the parameter space, which also occurs in splines on triangulations (Lai and Schumaker, 2007), is commonly known as the curse of dimensionality (CoD) by the tensor research community. CoD makes problems such as construction and evaluation of the tensor product B-spline Equation (5) intractable. Regardless, for functions which are expressible by a sum of a few separable terms (Equation 1), i.e.,


$$f\left(\text{x}\right)=\overset{R}{\underset{r=1}{\sum }}\left(\overset{D}{\underset{d=1}{\prod }}\phi_{r}^{\left(d\right)}\left(x_{d}\right)\right),$$

approximation by the expansion (Equation 5) is grossly inefficient. For such functions, the tensor C in Equation (5) is of low rank and has a more concise parameterization in the form of a polyadic decomposition into *R* terms:


(6) $$\begin{array}{l} C\left(\Gamma^{\left(1\right)},\ldots ,\Gamma^{\left(D\right)}\right)=〚\Gamma^{\left(1\right)},\ldots ,\Gamma^{\left(D\right)}〛=\overset{R}{\underset{r=1}{\sum }}\gamma_{r}^{\left(1\right)}⊗\cdots ⊗\gamma_{r}^{\left(D\right)}, \end{array}$$

in which Γ^(*d*)^ are the factor matrices of the CPD. To confirm that such a low-rank parameterization is actually possible, one simply has to approximate each component function $\phi_{r}^{\left(d\right)}$ in Equation (1) by a B-spline


(7) $$\begin{array}{l} \phi_{r}^{\left(d\right)}\left(x_{d}\right)\approx {\text{B}}^{\left(d\right)}\left(x_{d}\right)\gamma_{r}^{\left(d\right)}. \end{array}$$

Substituting (Equation 7) into (Equation 1) results then into the more compact approximant


(8) $$\begin{array}{l} \hat{f}(x;\;\Gamma^{\left(1\right)},\ldots ,\;\Gamma^{\left(D\right)})=C(\Gamma^{\left(1\right)},\ldots ,\\ \Gamma^{\left(D\right)})\cdot_{1}B^{\left(1\right)}(x_{1})\cdot_{2}\cdots \cdot_{D}B^{\left(D\right)}(x_{D}), \end{array}$$

where $C:{\mathbb{R}}^{\left(M_{1}+1\right)\times R}\times \cdots \times {\mathbb{R}}^{\left(M_{D}+1\right)\times R}\to {\mathbb{R}}^{\left(M_{1}+1\right)\times \cdots \times \left(M_{D}+1\right)}$ is now described by the polyadic decomposition (Equation 6). Indeed, in comparison to Equation (5), the approximant (Equation 8) can have orders of magnitude fewer parameters if *R* is low, i.e., less than $R\left(\sum_{d=1}^{D}M_{d}+1\right)$ parameters will be needed.

The ability to approximate functions efficiently through Equation (8) rests on the assumption that the number of separable terms *R* in Equation (1) are small. Classical results in approximation theory reveal that the level of difficulty in approximating a function can be characterized roughly by its degree of smoothness. Specifically, the rate at which a multivariate function can be approximated by a tensor product expansion of an orthogonal (polynomial) basis is proportionally tied to how many continuous derivatives the function possesses (measured in the Sobolev norm) (Mhaskar and Pai, 2000). Subsequently, a very smooth function which has a commensurate number of continuous derivatives with respect to the number of variables can be very compactly approximated with a few terms in Equation (1). However, such an analysis does not describe the whole picture at all.

The smoothness of a function does not alone capture the “rank of a function.” The class of functions well representable through Equation (1) is much richer, as a function can be easily nonsmooth but still of low rank; for example, any function of a sum of separable terms in which several of the component functions are nonsmooth. The work in Beylkin and Mohlenkamp (2005), Boussé et al. (2017), and Khoromskij (2018) discusses some interesting mechanisms under which multivariate functions can have good low-rank approximations, which are, for instance, achieved through sums of exponentials or sinc functions. At a very intuitive level, the low-rankness of a function captures the low complexity of a function. The bottom line is that negative results in DeVore et al. (1989) and Yarotsky (2017) (see introduction) put a fundamental “speed limit” to general robust approximation of a function, which is exponential in the number of dimensions, but simultaneously attenuated by the degree of smoothness. Any approach more effective must somehow utilize some additional structure in some way. In the case of Equation (8), this additional structure comes in the form of a low-rank tensor, which forms the basis of the success of tensor-based scientific computing (Hackbusch, 2012; Grasedyck et al., 2013; Khoromskij, 2018).

## 3. Regression

In a regression context, the aim is to learn a function $\hat{f}\left(\text{x}\right)$ as defined in Equation (8) that approximates the unknown target function *f* ∈ *C*([0, 1]^*D*^) defined on a *D*-dimensional feature space, from a set of *I* samples ${\left\{\left({\text{x}}_{i},y_{i}\right)\right\}}_{i=1}^{I}\subset {\left[0,1\right]}^{D}\times \mathbb{R}$. To achieve this, we minimize the quadratic objective


(9) $$\begin{array}{l} Q\left(\Gamma^{\left(1\right)},\ldots ,\Gamma^{\left(D\right)}\right): =\frac{1}{2}\overset{I}{\underset{i=1}{\sum }}{\left(\hat{f}\left({\text{x}}_{i};\Gamma^{\left(1\right)},\ldots ,\Gamma^{\left(D\right)}\right)-y_{i}\right)}^{2}. \end{array}$$

This problem can be interpreted in two ways as discussed in Subsection 3.1. Next, we derive the GN-based algorithm that exploits the low-rank and B-spline structure. Technical details have been moved to Appendix A for readability. To conclude this section, the practical feasibility of the proposed combination of low-rank and B-spline structure is illustrated numerically using synthetic and real-life data in Section 3.3.

### 3.1. Two Interpretations

While a GN-based algorithm is derived in the next subsection, it can be fruitful to discuss links with two other frameworks. First, the objective function (Equation 9) can be seen as a linear system of which the solution has a CPD constraint, i.e.,


$$\begin{array}{l} \text{A}\text{c}=\text{y}, \end{array}$$

in which the solution $\text{c}=\text{vec}\left(C\right)=\text{vec}\left(〚\Gamma^{\left(1\right)},\ldots ,\Gamma^{\left(D\right)}〛\right)$, and $\text{y}={\left[\begin{array}{ccc} y_{1} & \ldots & y_{I} \end{array}\right]}^{⊤}$. Using the definition of the mode-*d* tensor-matrix product, we can see that a row ${\text{a}}_{i}^{⊤}$ in $\text{A}\in {\mathbb{R}}^{I\times \prod_{d=1}\left(M_{d}+1\right)}$ is given by


(10) $$\begin{array}{l} {\text{a}}_{i}^{⊤}=\overset{D}{\underset{d=1}{⊗}}{\text{B}}^{\left(d\right)}\left(x_{i,d}\right) \end{array}$$

in which ⊗ is the Kronecker product and ${\text{B}}^{\left(d\right)}\left(x_{i,d}\right)$ is a row vector of evaluated B-splines in point *x*_*i,d*_. While a general solver such as Boussé et al. (2018) can be used, exploiting the Kronecker structure is crucial for achieving an efficient algorithm (see, e.g., Hendrikx et al., 2019) for a polynomial example that also involves symmetry. As Equation (10) does not involve symmetry, a new algorithm is derived here by exploiting the Kronecker structure as well as the B-spline basis structure.

Second, problem (Equation 9) can be seen as a decomposition of a *D*th-order incomplete tensor Y in which the *d*th dimension *J*_*d*_ is the number of unique values *x*_*i,d*_ over all samples *i*, hence *J*_*d*_ ≤ *I*. The number of known entries in Y is equal to *I*. By denoting the evaluated spline basis matrices **B**^(*d*)^ in these unique values *x*_*i,d*_ by ${\hat{\text{B}}}^{\left(d\right)}$ and by using multilinear calculus to rewrite (Equation 8), an incomplete tensor fitting problem is obtained in which a linear constraint is imposed on each factor matrix:


(11) $$\begin{array}{l} \underset{{\left\{\Gamma^{\left(d\right)}\right\}}_{d=1}^{D}}{\min}\frac{1}{2}{\left\|S*\left(Y-〚{\hat{\text{B}}}^{\left(1\right)}\Gamma^{\left(1\right)},{\hat{\text{B}}}^{\left(2\right)}\Gamma^{\left(2\right)},\ldots ,{\hat{\text{B}}}^{\left(D\right)}\Gamma^{\left(D\right)}〛\right)\right\|}_{\text{F}}^{2}, \end{array}$$

where S is a binary sampling tensor which is one if the entry is known or sampled, and zero otherwise. A GN algorithm for solving problem (Equation 11) is presented in Vervliet et al. (2017). In this paper, we improve upon this result by further exploiting the sparsity of the basis matrices thanks to the B-spline structure.

### 3.2. Gauss–Newton Algorithm

A basic descent algorithm to solve (Equation 9) is the alternating least squares (ALS) scheme (Harshman, 1970). In ALS, one exploits the multilinear structure (Equation 6) by freezing all but one of the factor matrices, so that the problem essentially becomes linear at each sub-iteration. Alternatively one may also approach the problem using a GN procedure, which has been shown to have superior convergence properties for tensor related problems with respect to ALS (see, e.g., Sorber et al., 2013; Vervliet et al., 2017; Vervliet and De Lathauwer, 2019).

In this section, we derive the necessary ingredients—the gradient ${\text{g}}_{r}^{\left(d\right)}$ and the GN step ${\text{p}}_{r}^{\left(d\right)}$, *r* = 1, …, *R*, and *d* = 1, …, *D*—for a GN type algorithm for solving (Equation 9). The dogleg trust region approach is used to ensure global convergence as this has proven successful in a tensor context (Sorber et al., 2013; Vervliet and De Lathauwer, 2019). As the dogleg algorithm is a standard algorithm in optimization—see, e.g., Nocedal and Wright (2006)—we assume variables $\gamma_{r}^{\left(d\right)}$ are updated from iteration *l* to *l* + 1 as


$$\begin{array}{l} \gamma_{r,l+1}^{\left(d\right)}\leftarrow \gamma_{r,l}^{\left(d\right)}+{\text{u}}_{r,l}^{\left(d\right)},\;r=1,\ldots ,R,\;d=1,\ldots ,D, \end{array}$$

in which ${\text{u}}_{r,l}^{\left(d\right)}$ is the dogleg step. (In the remainder, the iteration subscript *l* is dropped for simplicity of notation).

To show that the GN approximation is an appropriate choice for solving problem (Equation 9), consider first the Newton step ${\tilde{\text{p}}}_{r}^{\left(d\right)}$, which is obtained by solving the linear system of equations


(12) $$\begin{array}{l} \overset{D}{\underset{\tilde{d}=1}{\sum }}\overset{R}{\underset{\tilde{r}=1}{\sum }}\left(\frac{\partial^{2}Q\left(\Gamma^{\left(1\right)},\ldots ,\Gamma^{\left(D\right)}\right)}{\partial \gamma_{r}^{\left(d\right)}\partial \gamma_{\tilde{r}}^{\left(\tilde{d}\right)}}\right){\tilde{\text{p}}}_{\tilde{r}}^{\left(\tilde{d}\right)}=-\frac{\partial Q\left(\Gamma^{\left(1\right)},\ldots ,\Gamma^{\left(D\right)}\right)}{\partial \gamma_{r}^{\left(d\right)}}\\ \;=-{\text{g}}_{r}^{\left(d\right)}, \end{array}$$

for *r* = 1, …, *R* and *d* = 1, …, *D*. The left-hand-side matrix is the Hessian of Equation (9), while the right-hand side is the gradient ${\text{g}}_{r}^{\left(d\right)}$. Because of the low-rank structure in the coefficient tensor C, both the gradient and Hessian terms in Equation (12) are highly structured. A meticulous derivation of these quantities allows us to express them concisely. Let us define


$$\begin{array}{l} v_{r;i}^{\left(d\right)}=v_{r}^{\left(d\right)}\left({\text{x}}_{i};\Gamma^{\left(1\right)},\ldots ,\Gamma^{\left(D\right)}\right):=\overset{D}{\underset{k=1,k\neq d}{\prod }}{\text{B}}^{\left(k\right)}\left(x_{i,k}\right)\gamma_{r}^{\left(k\right)},\\ w_{r,\tilde{r};i}^{\left(d,\tilde{d}\right)}=w_{r,\tilde{r}}^{\left(d,\tilde{d}\right)}\left({\text{x}}_{i};\Gamma^{\left(1\right)},\ldots ,\Gamma^{\left(D\right)}\right):=\{\begin{array}{cc} 0 & d=\tilde{d}\\ \psi^{\left(d,\tilde{d}\right)}\left({\text{x}}_{i};\Gamma^{\left(1\right)},\ldots ,\Gamma^{\left(D\right)}\right) & r=\tilde{r}\\ 0 & r\neq \tilde{r} \end{array},\\ \psi_{i}^{\left(d,\tilde{d}\right)}=\psi^{\left(d,\tilde{d}\right)}\left({\text{x}}_{i};\Gamma^{\left(1\right)},\ldots ,\Gamma^{\left(D\right)}\right):=\overset{D}{\underset{k=1,k\neq d,\tilde{d}}{\prod }}{\text{B}}^{\left(k\right)}\left(x_{i,k}\right)\gamma_{\tilde{r}}^{\left(k\right)}. \end{array}$$

The gradient and Hessian terms can then be expressed as


(13) $$\begin{array}{l} \frac{\partial Q\left(\Gamma^{\left(1\right)},\ldots ,\Gamma^{\left(D\right)}\right)}{\partial \gamma_{r}^{\left(d\right)}}=\overset{I}{\underset{i=1}{\sum }}\eta_{i}v_{r;i}^{\left(d\right)}{{\text{B}}^{\left(d\right)}}^{⊤}\left(x_{i,d}\right), \end{array}$$

(14) $$\begin{array}{l} \frac{\partial^{2}Q\left(\Gamma^{\left(1\right)},\ldots ,\Gamma^{\left(D\right)}\right)}{\partial \gamma_{r}^{\left(d\right)}\partial \gamma_{\tilde{r}}^{\left(\tilde{d}\right)}}=\overset{I}{\underset{i=1}{\sum }}\xi_{i}\left(v_{r;i}^{\left(d\right)}{{\text{B}}^{\left(d\right)}}^{⊤}\left(x_{i,d}\right)\right)\left(v_{\tilde{r};i}^{\left(\tilde{d}\right)}{\text{B}}^{\left(\tilde{d}\right)}\left(x_{i,\tilde{d}}\right)\right)\\ \;+\eta_{i}w_{r,\tilde{r};i}^{\left(d,\tilde{d}\right)}{{\text{B}}^{\left(d\right)}}^{⊤}\left(x_{i,d}\right){\text{B}}^{\left(\tilde{d}\right)}\left(x_{i,\tilde{d}}\right), \end{array}$$

in which η_*i*_ ∈ ℝ is the residual, and ξ_*i*_ ∈ ℝ is a weight term:


(15) $$\begin{array}{l} \eta_{i}=\eta \left({\text{x}}_{i},y_{i};\Gamma^{\left(1\right)},\ldots ,\Gamma^{\left(D\right)}\right):=\hat{f}\left({\text{x}}_{i};\Gamma^{\left(1\right)},\ldots ,\Gamma^{\left(D\right)}\right)-y_{i}, \end{array}$$

(16) $$\begin{array}{l} \xi_{i}:=1. \end{array}$$

(The weight ξ_*i*_ will be important for the classification case; see Section 4.) Note that Equation (13) and (14) consist of only *I* terms, i.e., the number of samples.

In the GN procedure, the second term in the Hessian is dropped so that Equation (14) reduces to the Gramian of the Jacobian:


(17) $$\begin{array}{l} {\text{G}}_{r,\tilde{r}}^{\left(d,\tilde{d}\right)}: =\overset{I}{\underset{i=1}{\sum }}\xi_{i}\left(v_{r;i}^{\left(d\right)}{{\text{B}}^{\left(d\right)}}^{⊤}\left(x_{i,d}\right)\right)\left(v_{\tilde{r};i}^{\left(\tilde{d}\right)}{\text{B}}^{\left(\tilde{d}\right)}\left(x_{i,\tilde{d}}\right)\right). \end{array}$$

This Gramian can be a good approximation of the Hessian as the second term depends on the residual η_*i*_, which becomes small near a global optimum and zero if an exact solution exists, i.e., if *Q*(Γ^(1)^, …, Γ^(*D*)^) = 0. Moreover, the term $w_{r,\tilde{r};i}^{\left(d,\tilde{d}\right)}$ is often zero. The Gramian is positive semidefinite and allows for a locally convex approximation of the objective function. By dropping the second term in Equation (14), the system


(18) $$\begin{array}{l} \overset{D}{\underset{\tilde{d}=1}{\sum }}\overset{R}{\underset{\tilde{r}=1}{\sum }}{\text{G}}_{r,\tilde{r}}^{\left(d,\tilde{d}\right)}{\text{p}}_{\tilde{r}}^{\left(\tilde{d}\right)}=-\frac{\partial Q\left(\Gamma^{\left(1\right)},\ldots ,\Gamma^{\left(D\right)}\right)}{\partial \gamma_{r}^{\left(d\right)}},\\ \;r=1,\ldots ,R,\;d=1,\ldots ,D, \end{array}$$

is solved in lieu of Equation (12).

There are several provisions taken to solve the linear system (Equation 18) in an effective manner. Similar to earlier work (Sorber et al., 2013; Vervliet and De Lathauwer, 2019), system (Equation 18) is solved iteratively using the conjugate gradient (CG) method. The CG matrix only requires evaluations of matrix-vector products—in Equation (18), the matrix is the Gramian—which can be computed efficiently by exploiting the structure in the problem in analogous way as done in Hendrikx et al. (2019) and Vervliet et al. (2017); see Appendix A for the exact algorithmic details.

While CG requires a positive definite matrix, the method works for the positive semidefinite Gramian as well in practice, as both the starting point—the Cauchy point—for the CG method and the right hand side of Equation (18) are both orthogonal to the null space of the Gramian. As the trust-region approach ensures sufficient decrease, which is needed for convergence of the GN method, the number of (inner) CG iterations can be limited, lowering the computational cost further.

A key benefit of using splines is that the Gramian (Equation 18) enjoys an additional sparsity structure on top of the multilinear structure already caused by CPD constraint (Equation 6). If one exploits the sparsity pattern in the spline basis (recall that, unlike polynomials, the spline basis elements are compactly supported), the time complexity for evaluating the gradient and Gramian-vector product are both O(INRD) flop. Herein, $N:=\max_{d}N_{d}$ denotes the maximum order of the splines used in Equation (8). In practice, *N* is intentionally kept low (typically *N* = 4) in order to avoid usage of high-degree polynomials in the approximation. The complexity then effectively reduces down to O(IRD) flop. These savings are significant if one takes into consideration the maximum number of spline coefficients in the univariate component functions, i.e., $M:=\max_{d}M_{d}$. A failure to take advantage of the sparsity structure would have increased the complexity to O(IMRD) flop. This illustrates another advantage of B-splines over pure polynomials as there is no sparsity pattern to be exploited in the latter case.

### 3.3. Numerical Examples

A Matlab implementation of the proposed GN algorithm for the objective function (Equation 9) has been made. For the tensor and optimization related functions we use Tensorlab 3.0 (Vervliet et al., 2016). Next, we cover some interesting examples that examine the behavior of the proposed regression framework.

#### 3.3.1. Case Study I

We will start off with a function which is nonsmooth but of low rank, i.e.,


(19) $$\begin{array}{l} f\left(\text{x}\right)=|x_{1}||x_{2}|+\sin\left(2\pi x_{1}\right)\cos\left(2\pi x_{2}\right)+x_{1}^{2}x_{2},\\ \text{x}\in \left[-1,1\right]\times \left[-1,1\right]. \end{array}$$

**Figure 2:**
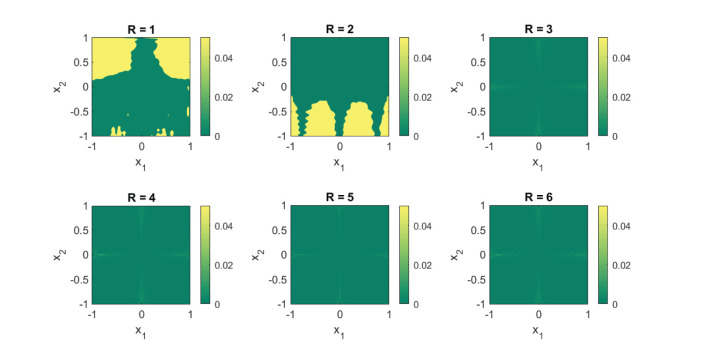
A *R* = 3 separable expansion is necessary to obtain a fit that is capable of capturing the global features of the function (Equation 19). We used 8,000 random function samples for our training set. The fits were obtained with a B-spline basis comprising of a uniformly distributed knot set and *M* = 35, *N* = 4. The plots depict the absolute error between the true function (Equation 19) and the obtained approximation using (Equation 8).

The function (Equation 19) has a kink on the lines *x*_1_ = 0 and *x*_2_ = 0 which is caused by the first separable term |*x*_1_||*x*_2_|. Despite of its nonsmooth features, it is still of low rank, particularly *R* = 3. Subsequently, Equation (19) can be well approximated with O(M) terms using Equation (8), instead of the $O\left(M^{2}\right)$ terms needed with Equation (5). In Figure 2 we actually demonstrate this numerically. The figure contains a plot of the absolute error between the obtained approximant (Equation 8) by solving (Equation 9) using the GN algorithm with random initialization and the true function (Equation 19). For our experiment, we took 8,000 random samples of the function uniformly over the domain for our training set. For each of the component functions (Equation 7), we chose a uniform knot distribution with *M* = 31 and *N* = 4. From the plots, one can clearly see that the first two low-rank approximations (i.e., *R* = 1, and *R* = 2) are of poor quality. This is expected, since a rank-one or a rank-two approximation cannot capture the full complexity of the function. In fact, the results will not even improve if more data samples are taken and the number of knots are increased for each of the component functions (Equation 7). The rank-three approximation is on the other hand of significantly better quality. This is expected because the true function (Equation 19) also contains three separable terms. Furthermore, the higher-rank approximations with *R* > 3 do not seem to add much value to the approximation. This also exhibits the property that the rank can be overestimated without loss in quality.

**Figure 3:**
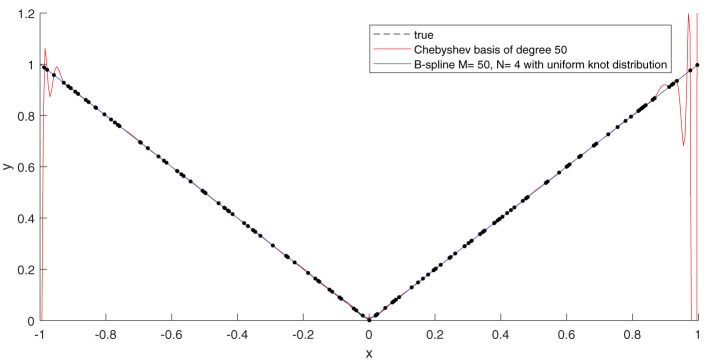
If an ordinary least-squares objective function is used to construct an approximation, high-degree polynomials become more susceptible to the Runge's phenomenon, resulting in wild oscillations near the boundary of the approximation interval. By keeping the order of the B-splines low, the Runge's effects can be circumvented. Shown in the plot is a least-squares fit of the function *f*(*x*) = |*x*| on the interval [−1, 1]. We used 160 random function samples (using a uniform distribution) to perform the regression. The samples are plotted in black dots.

**Figure 4:**
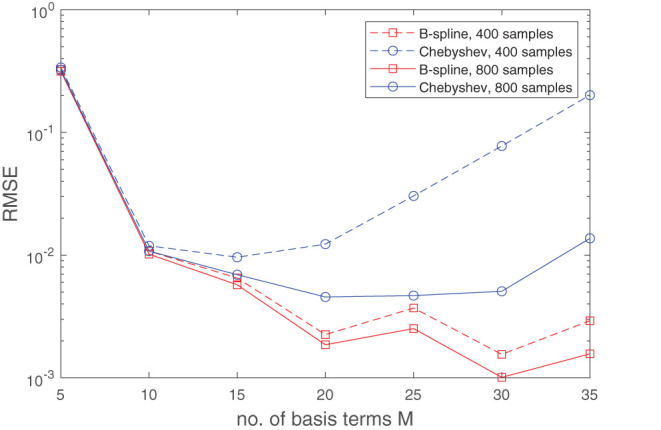
Runge's effects, as illustrated graphically in Figure 3, resurface again when the function (Equation 19) is approximated with a sum of separable terms (Equation 8) where the B-spline basis is replaced with a Chebyshev basis. This occurs due to the non-analytic term |*x*_1_||*x*_2_| which makes (Equation 19) non-differentiable on the lines *x*_1_ = 0 and *x*_2_ = 0. If a fit is generated through minimizing the least-squares objective function (Equation 9), a Chebyshev basis will, unlike for B-splines, start exhibiting degrading performance when the number of basis terms becomes large with respect to the size of the dataset. This can be observed in the plot above, which shows the median RMSE obtained over 50 experiment, in each of which a different random set of function samples (derived from a uniform distribution) were used. For the experiments, the B-spline basis comprised of a uniformly distributed knot set. The oscillations in RMSE curve for the B-spline basis can be attributed to the fact that the knots will not always lie exactly on the non-differentiable region of the function.

**Figure 5:**
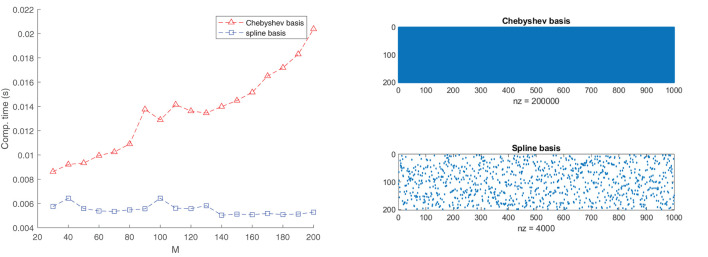
The exploitation of the sparsity in the B-spline basis matrix ${\text{A}}^{\left(d\right)}:=\left[\begin{array}{ccc} {{\text{B}}^{\left(d\right)}}^{⊤}\left(x_{1,d}\right) & \cdots & {{\text{B}}^{⊤}}^{\left(d\right)}\left(x_{I,d}\right) \end{array}\right]$ significantly accelerates the GN step. The left plot shows the average required computation time to pass through one cycle of the GN algorithm for a B-spline basis of order *N* = 4 and a Chebyshev basis. We used 1,000 random data points to fit the function (Equation 19). The rank is set to *R* = 3. The right plots provide a glimpse of the sparsity structure of the matrix **A**^(*d*)^ for a specific *d*. This sparsity is exploited in the implementation. In the case of the Chebyshev basis, there is no sparsity pattern to be exploited.

To test the effectiveness of using B-splines, we compared our results with an approximation using Chebyshev polynomials. It is well-known that the function *f*(*x*) = |*x*| can be easily subjugated to the Runge phenomenon. Without delving too much into the technical details, bad fits can particularly arise if the points picked are uniformly distributed and the degree of the polynomials is close to the number of function samples; see Figure 3. Intuitively, one would expect that Runge's effects would re-appear also in the fit of Equation (19). In Figure 4, this is actually confirmed. Apart from the quality of approximation, Figure 5 displays the average computation time required to pass through a single iteration of the GN algorithm for the B-spline basis as well as the Chebyshev basis. From the figure it can be clearly deduced that the computation time grows proportionally with the size of the Chebyshev basis, whereas it remains constant for the spline basis if only the number of knots are increased.

#### 3.3.2. Case Study II

The next example which we consider looks very similar to our first example, the only exception being that the first separable term is “rotated” by 45 degrees:


(20) $$\begin{array}{l} f\left(\text{x}\right)=\left|\frac{\sqrt{2}}{2}x_{1}-\frac{\sqrt{2}}{2}x_{2}\right|\left|\frac{\sqrt{2}}{2}x_{1}+\frac{\sqrt{2}}{2}x_{2}\right|+\sin\left(2\pi x_{1}\right)\cos\left(2\pi x_{2}\right)\\ \;+x_{1}^{2}x_{2},\;\text{x}\in \left[-1,1\right]\times \left[-1,1\right]. \end{array}$$

**Figure 6:**
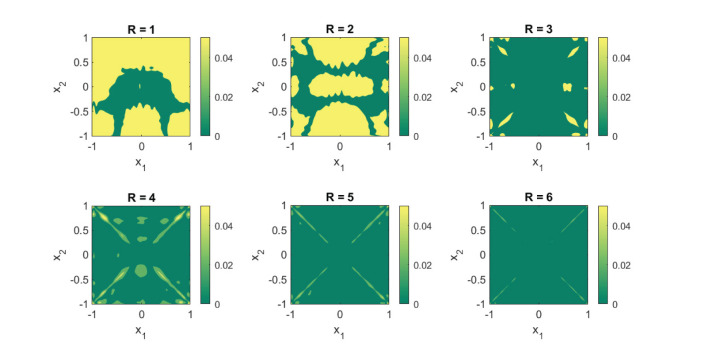
Replacing the term |*x*_1_||*x*_2_| in Equation (19) with $\left|\frac{\sqrt{2}}{2}x_{1}-\frac{\sqrt{2}}{2}x_{2}\right|\left|\frac{\sqrt{2}}{2}x_{1}+\frac{\sqrt{2}}{2}x_{2}\right|$ destroys the rank-three structure of Equation (19). Yet, an *R* = 6 separable expansion is sufficient to get a an approximation that is capable of capturing the global features of Equation (20). We used 8,000 random function samples for our training set. The fits were obtained with a B-spline basis comprising of a uniformly distributed knot set and *M* = 35, *N* = 4. The plots show the absolute error between the true function (Equation 19) and the obtained approximation using (Equation 8).

This change is significant, however, since the rotation breaks the rank-one structure of the first term. Unlike our previous example, the function (Equation 20) has rank strictly greater than *R* = 3. Given that the nonsmooth portion of the function is no longer aligned with the coordinates *x*_1_ and *x*_2_, one would expect that an approximation with Equation (8) would do quite poorly. In comparison to Case study I, this is also the case. Figure 6 displays the obtained results for different rank values using the exact same configuration as in Figure 2. The absolute errors are clearly larger. Surprisingly, however, the global trends of the function are still well-captured with a rank *R* = 3 approximation. Furthermore, a rank *R* = 6 approximation is of comparable quality to its counterpart of Figure 2. The results indicate that, even if a function may be mathematically of high rank, good approximations that capture the global features of the function can already be achieved with relatively few rank-one terms.

#### 3.3.3. Case Study III: NASA Airfoil Self Noise

**Figure 7:**
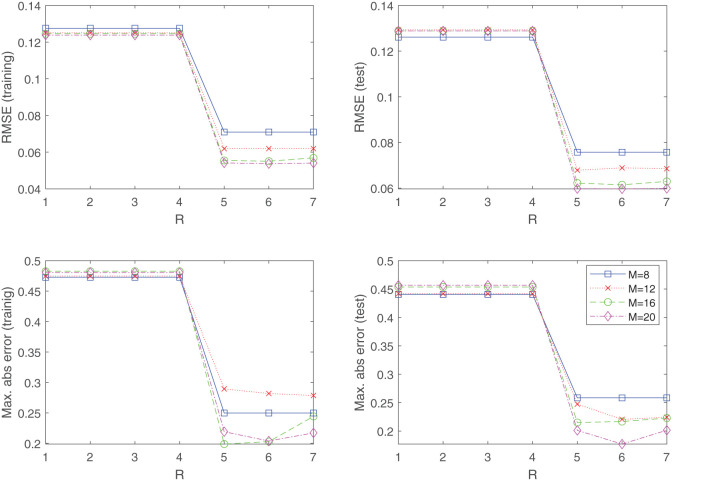
The NASA dataset of the UCI machine learning repository is well modeled by a *R* = 5 separable function, where we see a sudden drop in the training and test error. This indicates the benefits of using (Equation 8) over a direct tensor product expansion (Equation 5), where no low-rank structures are exploited. For generating the results, the knot distribution is kept uniform for all experiments.

The final example which we consider is the task of fitting an experimentally obtained dataset. We examine the NASA dataset (Brooks et al., 1989) obtained from the UCI machine learning repository (Dua and Graff, 2019) which is comprised of data concerning different size NACA 0012 airfoils subjected to various wind tunnel speeds and angles of attack. The goal is to model the sound, or self noise generated by the airfoil, as a function of the frequency, angle of attack, chord length, free-stream velocity, and the suction-side displacement thickness. The dataset has been normalized and contains 1,503 measurements in total. We randomly split the dataset into a training dataset (1,202 samples) and a test dataset (301 samples). Figure 7 displays the obtained results by using the approximant (Equation 8) to fit the data by means of minimizing the objective function (Equation 9). As in the previous examples, a uniformly distributed knot distribution was chosen for the component functions (Equation 7), and shown in Figure 7 is the root mean squared error (RMSE) and maximal absolute error obtained for various configurations. Generating a fit directly with Equation (5) would have been prohibitively expensive, since the dependent variable is a function of five variables. However, Figure 7 reveals that the target function possesses low-rank structure, and can be well modeled by *R* = 5 separable terms, where we see a sudden drop in the training and test error. Furthermore, it shows that the inclusion of any additional separable terms does not amount to significant performance improvements of the fit.

## 4. Classification

If the goal is to classify samples into two classes, other loss functions than least-squares (Equation 9) are more suitable. In Subsection 4.1, we show how the logistic loss function can be combined with B-splines and the low-rank approximation of the coefficient tensors following Equation (8). Next, the GN algorithm is generalized to accommodate this loss function in Subsection 4.2. Finally, the utility of low-rank approximations in combination with B-splines for classification problems is illustrated via numerical experiments.

### 4.1. Logistic Cost Function

A (binary) classification function defined on a *D*-dimensional feature space can be modeled as the map *g*:[0, 1]^*D*^ → {0, 1} given by


$$g\left(\text{x}\right)=\{\begin{array}{cc} 0 & f\left(\text{x}\right)\leq 0\\ 1 & f\left(\text{x}\right)>0 \end{array},$$

where *f* ∈ *C*([0, 1]^*D*^) is a continuous function whose zero level set partitions the domain into the two classification sets. Instead of enforcing a hard threshold between the two sets, one can smoothen this transition by replacing the step function with the logistic function σ_α_:*t* ↦ 1/(exp(−α*t*) + 1), i.e.


$$\begin{array}{l} g_{\alpha }\left(\text{x}\right): =\left(\sigma_{\alpha }◦f\right)\left(\text{x}\right)=\sigma_{\alpha }\left(f\left(\text{x}\right)\right), \end{array}$$

where the parameter α > 0 controls the gradient in the transition zone. To incorporate the low-rank constraint and the B-spline basis, the function *g*_α_ is replaced by the approximant


(21) $$\begin{array}{l} {ĝ}_{\alpha }\left(\text{x};\Gamma^{\left(1\right)},\ldots ,\Gamma^{\left(D\right)}\right):=\sigma_{\alpha }◦\hat{f}\left(\text{x};\Gamma^{\left(1\right)},\ldots ,\Gamma^{\left(D\right)}\right), \end{array}$$

where $\hat{f}$ refers again to Equation (8). Given a collection of data samples ${\left\{\left({\text{x}}_{i},y_{i}\right)\right\}}_{i=1}^{I}\subset {\left[0,1\right]}^{D}\times \left\{0,1\right\}$, where *y*_*i*_ ∈ {0, 1} denote the labels, the performance of a classifier ĝ_α_ can be characterized by the tendency of the quantity


$$\begin{array}{l} 0\leq \underset{y_{i}=0}{\prod }\left(1-{ĝ}_{\alpha }\left({\text{x}}_{i};\Gamma^{\left(1\right)},\ldots ,\Gamma^{\left(D\right)}\right)\right)\\ \;\underset{y_{i}=1}{\prod }{ĝ}_{\alpha }\left({\text{x}}_{i};\Gamma^{\left(1\right)},\ldots ,\Gamma^{\left(D\right)}\right)\leq 1, \end{array}$$

to lean toward unity. By applying a logarithmic transformation, the recovery of a good classifier ĝ_α_ can hence be obtained by minimizing the objective function


(22) $$\begin{array}{l} L_{\alpha }\left(\Gamma^{\left(1\right)},\ldots ,\Gamma^{\left(D\right)}\right):=-\overset{I}{\underset{i=1}{\sum }}y_{i}\log{ĝ}_{\alpha }\left({\text{x}}_{i};\Gamma^{\left(1\right)},\ldots ,\Gamma^{\left(D\right)}\right)\\ \;+\left(1-y_{i}\right)\log\left(1-{ĝ}_{\alpha }\left({\text{x}}_{i};\Gamma^{\left(1\right)},\ldots ,\Gamma^{\left(D\right)}\right)\right). \end{array}$$

### 4.2. Generalized Gauss–Newton Algorithm

Even though the GN algorithm is derived for least-squares problems, it can be generalized easily to accommodate other loss functions. Following general results in Schraudolph (2002) and for tensor decompositions (Vandecapelle et al., 2020; Vandecappelle et al., 2021), the generalized GN algorithm can be derived similarly to the strategy in Subsection 3.2. In the dogleg trust-region framework, the necessary GN direction ${\text{p}}_{r}^{\left(d\right)}$ is derived starting from the linear system:


$$\begin{array}{l} \overset{D}{\underset{\tilde{d}=1}{\sum }}\overset{R}{\underset{\tilde{r}=1}{\sum }}\left(\frac{\partial^{2}L_{\alpha }\left(\Gamma^{\left(1\right)},\ldots ,\Gamma^{\left(D\right)}\right)}{\partial \gamma_{r}^{\left(d\right)}\partial \gamma_{\tilde{r}}^{\left(d\right)}}\right){\text{p}}_{\tilde{r}}^{\left(\tilde{d}\right)}=-\frac{\partial L_{\alpha }\left(\Gamma^{\left(1\right)},\ldots ,\Gamma^{\left(D\right)}\right)}{\partial \gamma_{r}^{\left(d\right)}},\\ r=1,\ldots ,R,\;d=1,\ldots ,D, \end{array}$$

in which the right-hand side is the gradient and is given by


$$\begin{array}{l} \frac{\partial L_{\alpha }\left(\Gamma^{\left(1\right)},\ldots ,\Gamma^{\left(D\right)}\right)}{\partial \gamma_{r}^{\left(d\right)}}=\overset{I}{\underset{i=1}{\sum }}\eta_{i}v_{r;i}^{\left(d\right)}{{\text{B}}^{\left(d\right)}}^{⊤}\left(x_{i,d}\right), \end{array}$$

and the blocks of the Hessian in the left-hand side are given by


$$\begin{array}{l} \frac{\partial^{2}L_{\alpha }\left(\Gamma^{\left(1\right)},\ldots ,\Gamma^{\left(D\right)}\right)}{\partial \gamma_{r}^{\left(d\right)}\partial \gamma_{\tilde{r}}^{\left(\tilde{d}\right)}}=\overset{I}{\underset{i=1}{\sum }}\xi_{i}\left(v_{r;i}^{\left(d\right)}{{\text{B}}^{\left(d\right)}}^{⊤}\left(x_{i,d}\right)\right)\left(v_{\tilde{r};i}^{\left(\tilde{d}\right)}{\text{B}}^{\left(\tilde{d}\right)}\left(x_{i,\tilde{d}}\right)\right)\\ \;+\eta_{i}w_{r,\tilde{r};i}^{\left(d,\tilde{d}\right)}{{\text{B}}^{\left(d\right)}}^{⊤}\left(x_{i,d}\right){\text{B}}^{\left(\tilde{d}\right)}\left(x_{i,\tilde{d}}\right). \end{array}$$

The main difference with respect to the least-squares objective (Equation 9) is that the weights η_*i*_, ξ_*i*_ ∈ ℝ are now given by


(23) $$\begin{array}{l} \eta_{i}=\eta_{i}\left({\text{x}}_{i},y_{i};\Gamma^{\left(1\right)},\ldots ,\Gamma^{\left(D\right)}\right):=\alpha \left({ĝ}_{\alpha }\left({\text{x}}_{i};\Gamma^{\left(1\right)},\ldots ,\Gamma^{\left(D\right)}\right)-y_{i}\right), \end{array}$$

(24) $$\begin{array}{l} \xi_{i}=\xi_{i}\left({\text{x}}_{i};\Gamma^{\left(1\right)},\ldots ,\Gamma^{\left(D\right)}\right):=\alpha^{2}{ĝ}_{\alpha }\left({\text{x}}_{i};\Gamma^{\left(1\right)},\ldots ,\Gamma^{\left(D\right)}\right)\\ \;\left(1-{ĝ}_{\alpha }\left({\text{x}}_{i};\Gamma^{\left(1\right)},\ldots ,\Gamma^{\left(D\right)}\right)\right). \end{array}$$

Similar to Subsection 3.2, a GN type approximation to the Hessian can be made by neglecting the second term in Equation (24), resulting in the linear system


(25) $$\begin{array}{l} \overset{D}{\underset{\tilde{d}=1}{\sum }}\overset{R}{\underset{\tilde{r}=1}{\sum }}{\text{G}}_{r,\tilde{r}}^{\left(d,\tilde{d}\right)}{\text{p}}_{\tilde{r}}^{\left(\tilde{d}\right)}=-\frac{\partial L_{\alpha }\left(\Gamma^{\left(1\right)},\ldots ,\Gamma^{\left(D\right)}\right)}{\partial \gamma_{r}^{\left(d\right)}},\;r=1,\ldots ,R,\\ d=1,\ldots ,D, \end{array}$$

in which


$$\begin{array}{l} {\text{G}}_{r,\tilde{r}}^{\left(d,\tilde{d}\right)}: =\overset{I}{\underset{i=1}{\sum }}\xi_{i}\left(v_{r;i}^{\left(d\right)}{{\text{B}}^{\left(d\right)}}^{⊤}\left(x_{i,d}\right)\right)\left(v_{\tilde{r};i}^{\left(\tilde{d}\right)}{\text{B}}^{\left(\tilde{d}\right)}\left(x_{i,\tilde{d}}\right)\right). \end{array}$$

This is again a good approximation to the Hessian as η_*i*_ is small if the residual ${ĝ}_{\alpha }\left({\text{x}}_{i};\Gamma^{\left(1\right)},\ldots ,\Gamma^{\left(D\right)}\right)-y_{i}$ is small, and many of the values $w_{r,\tilde{r};i}^{\left(d,\tilde{d}\right)}=0$. As a consequence of the positivity of the weights (Equation 24), the Gramian (Equation 17) associated with the logistic objective is also positive semidefinite, and the CG method can again be used to solve the linear system (Equation 25) iteratively using only matrix-vector products.

### 4.3. Numerical Examples

The proposed generalized GN has been implemented in Matlab using Tensorlab 3.0 (Vervliet et al., 2016). We examine the behavior of the classification framework with some illustrative examples.

#### 4.3.1. Case Study IV

**Figure 8:**
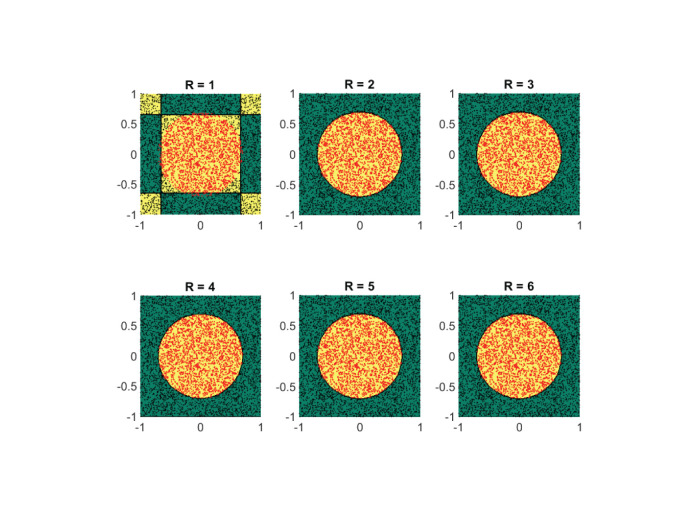
For a simple circular shaped classification set, two separable terms suffice for a good classification. Shown are the obtained results on a synthetic dataset by using the logistic loss function (Equation 22). The recovered classification sets are marked by the yellow and green contours, whereas the labeled data used for training are marked by red and black dots.

**Figure 9:**
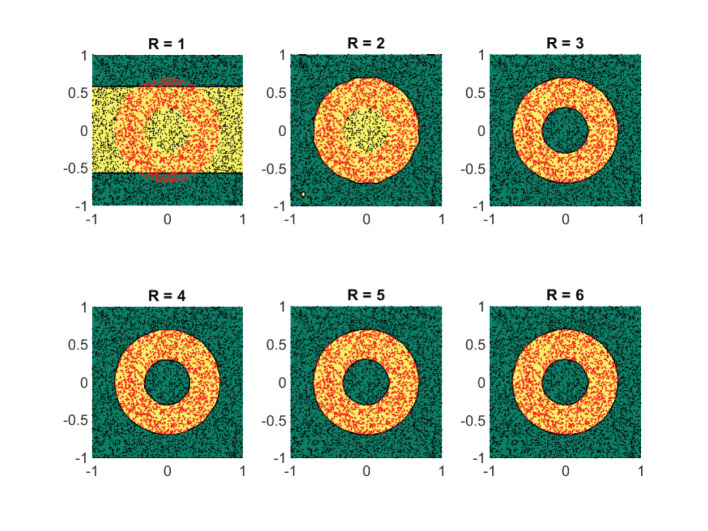
For a donut-shaped classification set, three separable terms are enough for a good classification. Shown are the obtained results on a synthetic dataset by using the logistic loss function (Equation 22). The recovered classification sets are marked by the yellow and green contours, whereas the labeled data used for training are marked by red and black dots.

**Figure 10:**
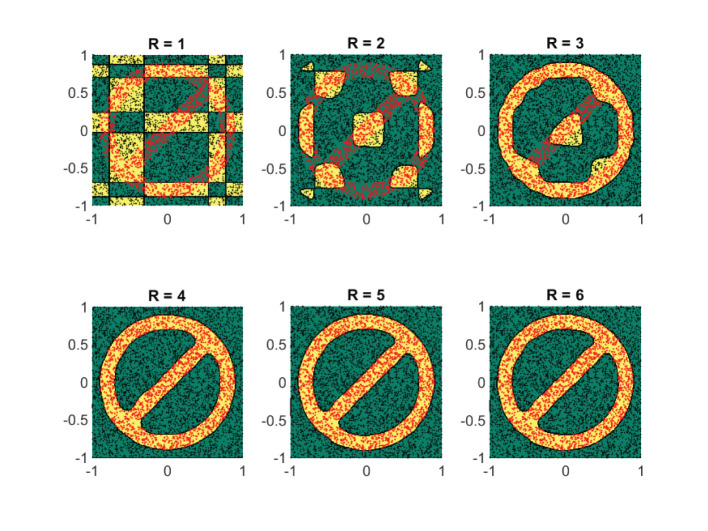
For a classification set shaped in the form of a no-entry sign, at least four separable terms are required for a good classification. Shown are the obtained results on a synthetic dataset by using the logistic loss function (Equation 22). The recovered classification sets are marked by the yellow and green contours, whereas the labeled data used for training are marked by red and black dots.

**Figure 11:**
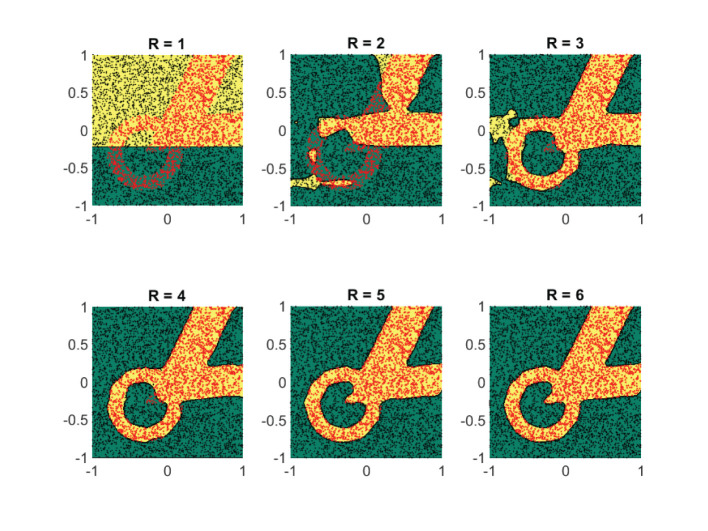
For this particular classification set, at least five separable terms are required for a decent classification. Shown are the obtained results on a synthetic dataset by using the logistic loss function (Equation 22). The recovered classification sets are marked by the yellow and green contours, whereas the labeled data used for training are marked by red and black dots.

At first, we assess the performance of the framework on some synthetically generated datasets. We consider classification sets that take on increasingly more complex geometric shapes on the two-dimensional plane. Since the geometric shapes are composed of simpler geometric operations, it is expected that a low-rank approximant of type (Equation 21) would be sufficient to obtain a decent separation between the two classes. In Figures 8–11, these expectations are confirmed, in which the classification sets are recovered by minimizing (Equation 22) for some randomly generated labeled data. The plots in the figures reveal that by increasing the number of separable terms in Equation (21), better classification can be achieved. The number of separable terms needed to get a decent separation between the two classes is roughly proportional to how “complex” the classification sets themselves are. The circular shaped set (Figure 8) required only *R* = 2 terms for a decent separation. From a theoretical standpoint, this is expected, since one can model a circular boundary by the level sets of the rank-two separable function


$$f\left(\text{x}\right)=e^{-\left(x_{1}^{2}+x_{2}^{2}\right)}-a=〚[\begin{array}{cc} e^{-x_{1}^{2}} & 1 \end{array}],[\begin{array}{cc} e^{-x_{2}^{2}} & -a \end{array}]〛.$$

On the other hand, the donut-shaped set (Figure 9) required *R* = 3 terms. Again, this is expected, since the donut-shaped set can be modeled through the level sets of the rank-three separable function


$$\begin{array}{l} f\left(\text{x}\right)=e^{-\left(x_{1}^{2}+x_{2}^{2}\right)}-e^{-b\left(x_{1}^{2}+x_{2}^{2}\right)}-a\\ \;=〚[\begin{array}{ccc} e^{-x_{1}^{2}} & e^{-bx_{1}^{2}} & 1 \end{array}],[\begin{array}{ccc} e^{-x_{2}^{2}} & e^{-bx_{2}^{2}} & a \end{array}]〛. \end{array}$$

The no-entry sign shaped classification set (Figure 10) and the final example (Figure 11) are more complex, but interestingly require only *R* = 4 and *R* = 5 terms, respectively. Similar to the regression problem, overestimation of the rank values do not seem to lead to a performance degradation.

**Figure 12:**
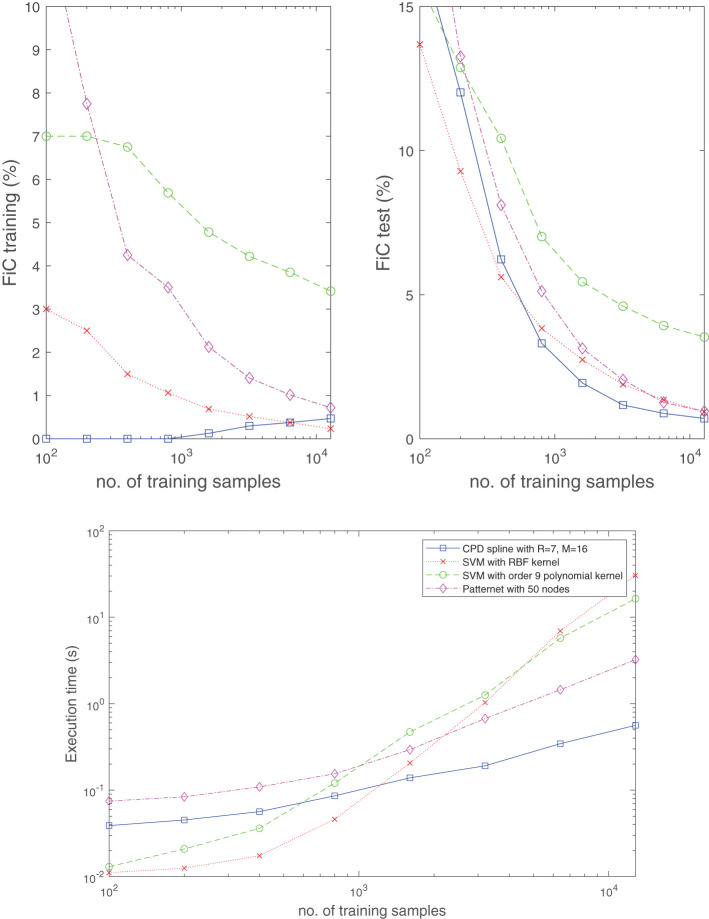
The classifier (Equation 21) is competitive with well-established techniques in terms of classification accuracy and computation time. Shown is the median result obtained from 50 randomly generated training and test datasets for the classification problem in Figure 11. Specifically, for the SVMs and NN, the built-in Matlab routines fitcsvm and patternnet were used to develop the results. Whereas the top two plots display the obtained fraction incorrect (FiC) for the training and test dataset, respectively, the bottom plot shows the required CPU time to complete the training. The test dataset size always equals 5000 samples.

In Figure 12, the performance of the classifier (Equation 21) is also compared with existing techniques such as support vector machines (SVMs) using an RBF or polynomial kernel, and a single hidden layer neural network (NN). By considering the classification problem in Figure 11 and taking the median result over 50 experiments (in each of which randomly generated training and test datasets were taken from a uniform distribution), the top two plots in Figure 12 show the obtained fraction incorrect (FiC) for the various methods as a function of the number of training samples. From the plots, it is evident that the classifier (Equation 21) with *R* = 7, *M* = 16 (224 parameters) consistently achieves lower training and test errors for this problem, when compared to a NN with 50 nodes (200 parameters) or an RBF-kernel-based SVM. These lower error rates are obtained with relatively shorter CPU time for completing the training. In particular, the required CPU time grows at a milder pace (with respect to the training dataset size) when compared to SVMs; see bottom plot in Figure 12. Another observation is that, in the case of the CPD spline model, the FiC for the training dataset slightly increases with the number of training samples. We associate this phenomena with overparameterization. For small datasets, it is always possible to find some fit for which all training samples are classified exactly. However, this does not imply that the classification has been done correctly, and this reflected in the poor FiC of the test dataset in those cases.

#### 4.3.2. Case Study V

**Figure 13:**
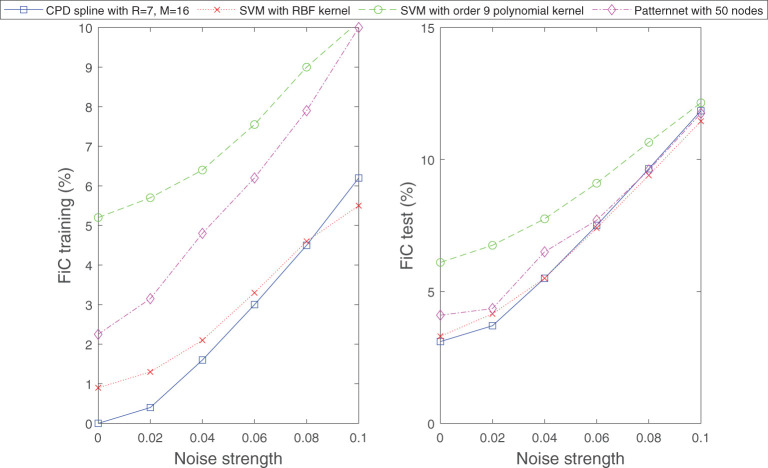
Similar to well-established techniques for classification, the classifier (Equation 21) is capable of separating classification sets with ambiguous boundaries. Shown are the obtained results for the classification problem considered in Figure 11, however, now the data samples are randomly perturbed by a vector of specific length (indicated by noise strength). The perturbation generates an ambiguous boundary between the two classes. The number of samples for the training and test dataset is set to 1000.

**Figure 14:**
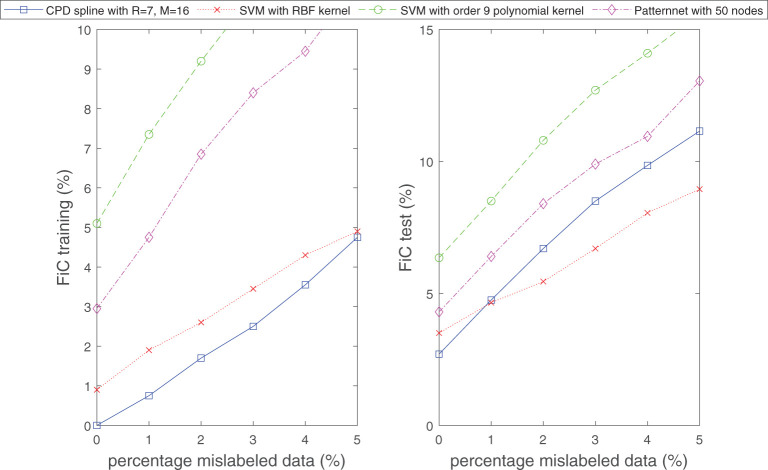
Similar to well-established techniques for classification, the classifier (Equation 21) is robust against mislabeled data. Shown are the obtained results for the classification problem considered in Figure 11, however, now in a situation where a particular percentage of data is intentionally mislabeled. The number of samples for the training and test dataset is set to 1,000.

In the next example, we subject the classification problem considered in Figure 11 to noise. We examine how the classifier (Equation 21) behaves if the boundary between the two classes is ambiguous or if a small random subset of the samples in both classes is intentionally mislabeled. Figures 13, 14 display the obtained results. From the figures it can be observed that under these noisy circumstances, the performance of Equation (21) degrades in the same rate as those of established methods of classification.

#### 4.3.3. Case Study VI: Banknote Authentication

**Figure 15:**
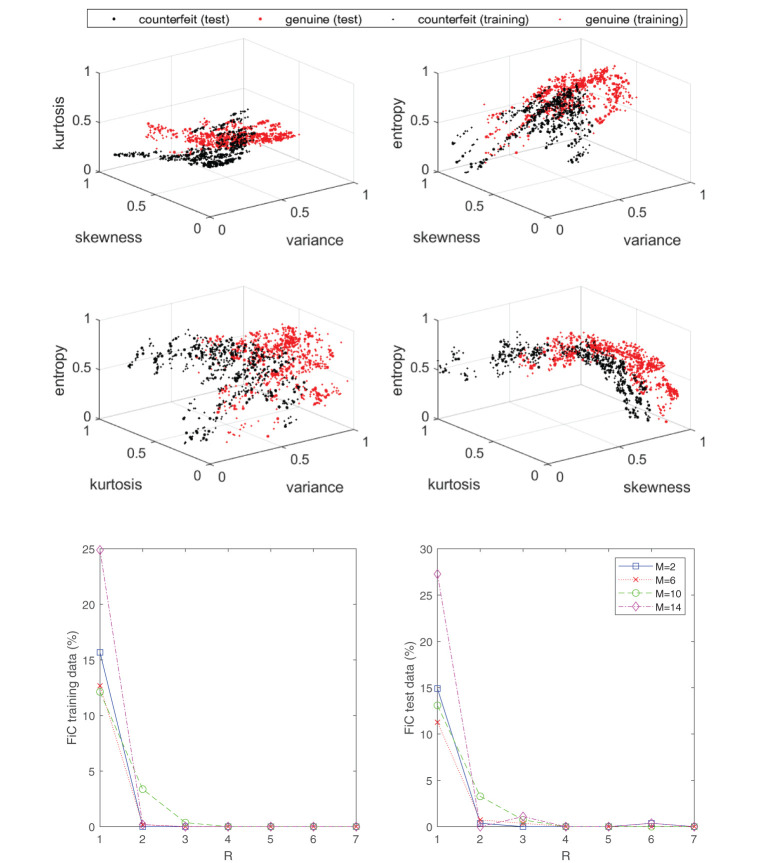
Even though the labeled data of the banknote problem live in a four-dimensional space, the classification problem can still be effectively “solved” with a low-rank classifier (Equation 21). Specifically, as seen in the bottom plot, an *R* = 2 separable expansion is sufficient to obtain a well-performing classifier with marginal error rates. The top four plots display the labeled data associated with the banknote dataset from the UCI machine learning repository.

The last example that we consider is the task of training a classifier to separate genuine and forged banknotes from each other. The UCI machine learning repository (Dua and Graff, 2019) contains a dataset where a collection of banknote images (forged and genuine) have been postprocessed in order to determine their variance, skewness, kurtosis, and entropy. These four features may be used to train a classifier. In Figure 15, the labeled dataset is displayed in the top four plots. The dataset has been normalized and split into a training set consisting of 1097 samples and a test dataset consisting of 275 samples. The bottom two plots in Figure 15 display the obtained results by using the approximant (Equation 21) to fit the data by means of minimizing the objective function (Equation 22). The classification error is shown for various configurations of *R* and *M*, which denote the number of rank-one and spline basis terms, respectively. A uniform knot distribution was chosen for the component functions (Equation 7) and the order is set to *N* = 4. From the plots it can be observed that as few as *R* = 2 separable terms are sufficient to get a complete separation between the two classes. In other words, the dataset may live in a higher-dimensional space (4D), but loosely speaking the classification problem is of comparable complexity with respect to the earlier synthetic example in Figure 8, which also only required two rank-one terms.

## 5. Conclusions and Future Work

We have introduced a supervised learning framework for regression and classification tasks which aims to approximate target functions with a sum of (few) separable terms. Each of the univariate component functions in the separable terms were discretized by a B-spline, resulting in an approximant where the underlying coefficient tensor of the tensor product expansion has a low rank polyadic decomposition parametrization. By taking advantage of the multilinear structure and the sparsity pattern of the compactly supported B-spline basis, a Gauss–Newton algorithm was introduced to train the model efficiently.

We have provided some illustrative examples which reveal the rationale behind the proposed framework and the low-rank mechanisms that are at play. In particular, the presented real-life experiments show that low-rank structures do appear in practice and using approximants of the type (Equation 8 or 21) do have their merit as they allow one to break the curse of dimensionality that is imposed by a direct tensor product B-spline basis (Equation 5). Henceforth, further development of this framework of supervised learning is worthwhile and could address more sophisticated, real-life machine learning problems.

There are several directions in which the present work can be extended or further explored. These include particularly matters such as appropriate (adaptive) knot selection heuristics. Furthermore, an extension to multiclass classification using softmax regression has not yet been considered to keep the current exposition concise, but is relatively straightforward to do. Finally, more complicated tensor decomposition architectures can be explored and further investigated. Particularly those of a hierarchical nature sound promising, given that many complex decision processes can be viewed as the consequence of smaller intermediate steps.

## Data Availability Statement

Publicly available datasets were analyzed in this study. This data can be found here: https://archive.ics.uci.edu/ml/index.php. The code used for the experiments and to generate the figures in this paper can be found on www.tensorlabplus.net.

## Author Contributions

NG and NV conjointly developed the theory and Matlab implementation. NG is the main contributor to the numerical experiments and also wrote the first draft of the manuscript. NV reworked the presentation of the algorithms. LD conceived the idea and supervised the project. All authors contributed to manuscript revision, read, and approved the submitted version.

## Funding

This work was supported by the Research Foundation Flanders (FWO) *via* projects G086518N, G086318N, and *via* postdoc grant 12ZM220N; KU Leuven Internal Funds *via* projects C16/15/059 and IDN/19/014; Fonds de la Recherche Scientifique—FNRS and the Fonds Wetenschappelijk Onderzoek—Vlaanderen under EOS project no. 30468160 (SeLMA). This research received funding from the Flemish Government under the Onderzoeksprogramma Artificiële Intelligentie (AI) Vlaanderen program.

## Conflict of Interest

The authors declare that the research was conducted in the absence of any commercial or financial relationships that could be construed as a potential conflict of interest.

## Publisher's Note

All claims expressed in this article are solely those of the authors and do not necessarily represent those of their affiliated organizations, or those of the publisher, the editors and the reviewers. Any product that may be evaluated in this article, or claim that may be made by its manufacturer, is not guaranteed or endorsed by the publisher.
