# Supplementary material for: Regression and Classification With Spline-Based Separable Expansions
Source: Front Big Data. 2022 Feb 11;5:688496. doi: 10.3389/fdata.2022.688496 (PMC8874272; doi:10.3389/fdata.2022.688496)
Supplement: Supplementary file 1 [file Presentation_1.pdf]

Supplementary Material

1 EVALUATION OF THE GRADIENT AND GRAMIAN-VECTOR PRODUCTS

The gradient and Gramian-vector products associated with the objective functions in sections 3 and 4 can be evaluated efficiently by exploiting both the multilinear structure resulting from the low-rank approximation of the coefficient tensor, and the compact support property of the B-spline basis functions. In this appendix, we first develop vectorized expressions for auxiliary variables. Next, these expressions are used to compute the gradient and Gramian-vector products. Finally, we discuss the complexity of the algorithm.

1.1 Vectorized expressions for the gradient and Gramian

To facilitate further computations, we first construct a matrix $\mathbf{A}^{(d)}$, $d = 1, \dots, D$, which contains the set of M B-spline basis functions evaluated in $\mathbf{x}_i$, $i = 1, \dots, I$ as its columns:

$$\mathbf{A}^{(d)} := \begin{bmatrix} {\mathbf{B}^{(d)}}^\top(x_{1,d}) & \cdots & {\mathbf{B}^{(d)}}^\top(x_{I,d}) \end{bmatrix} \in \mathbb{R}^{M \times I}.$$

The gradient $\mathbf{g}_r^{(d)} := \frac{\partial Q}{\partial \boldsymbol{\gamma}_r^{(d)}}$ in the regression case, or $\mathbf{g}_r^{(d)} := \frac{\partial L_\alpha}{\partial \boldsymbol{\gamma}_r^{(d)}}$ in the classification case, can then be expressed in vectorized form as:

$$\begin{aligned}
\mathbf{g}_r^{(d)} &= \sum_{i=1}^{I} \eta_i v_{r;i}^{(d)} {\mathbf{B}^{(d)}}^\top(x_{i,d}) \\
&= \begin{bmatrix} {\mathbf{B}^{(d)}}^\top(x_{1,d}) & \cdots & {\mathbf{B}^{(d)}}^\top(x_{I,d}) \end{bmatrix} \left(\begin{bmatrix} v_{r;1}^{(d)} \\ \vdots \\ v_{r;I}^{(d)} \end{bmatrix} * \begin{bmatrix} \eta_1 \\ \vdots \\ \eta_I \end{bmatrix} \right) \\
&= \begin{bmatrix} {\mathbf{B}^{(d)}}^\top(x_{1,d}) & \cdots & {\mathbf{B}^{(d)}}^\top(x_{I,d}) \end{bmatrix} \left(\begin{bmatrix} \prod_{k=1,k\neq d}^{D} \mathbf{B}^{(k)}(x_{i,k}) \boldsymbol{\gamma}_r^{(k)} \\ \vdots \\ \prod_{k=1,k\neq d}^{D} \mathbf{B}^{(k)}(x_{i,k}) \boldsymbol{\gamma}_r^{(k)} \end{bmatrix} * \begin{bmatrix} \eta_1 \\ \vdots \\ \eta_I \end{bmatrix} \right) \\
&= \mathbf{A}^{(d)} \left(\left(\mathop{\ast}_{k=1,k\neq d}^{D} {\mathbf{A}^{(k)}}^\top \boldsymbol{\gamma}_r^{(k)} \right) * \boldsymbol{\eta} \right).
\end{aligned}$$

Similarly, the required Gramian-vector products, i.e., the products of the Gramian built using blocks $\mathbf{G}_{r,\tilde{r}}^{(d,\tilde{d})}$, and the vector built from $\mathbf{z}_{\tilde{r}}^{(\tilde{d})}$, can be expressed as

$$\begin{aligned}
\mathbf{w}_r^{(d)} &:= \sum_{\tilde{d}=1}^{D}\sum_{\tilde{r}=1}^{R}\mathbf{G}_{r,\tilde{r}}^{(d,\tilde{d})}\mathbf{z}_{\tilde{r}}^{(\tilde{d})} \\
&= \sum_{\tilde{d}=1}^{D}\sum_{\tilde{r}=1}^{R}\left(\sum_{i=1}^{I}\xi_i\left(v_{r;i}^{(d)}\mathbf{B}^{(d)^\top}(x_{i,d})\right)\left(v_{\tilde{r};i}^{(\tilde{d})}\mathbf{B}^{(\tilde{d})}(x_{i,\tilde{d}})\right)\right)\mathbf{z}_{\tilde{r}}^{(\tilde{d})} \\
&= \sum_{\tilde{d}=1}^{D}\sum_{\tilde{r}=1}^{R}\sum_{i=1}^{I}\left(v_{r;i}^{(d)}\mathbf{B}^{(d)^\top}(x_{i,d})\right)\xi_i v_{\tilde{r};i}^{(\tilde{d})}\left(\mathbf{B}^{(\tilde{d})}(x_{i,\tilde{d}})\mathbf{z}_{\tilde{r}}^{(\tilde{d})}\right) \\
&= \sum_{\tilde{d}=1}^{D}\sum_{\tilde{r}=1}^{R}\left[v_{r;1}^{(d)}\mathbf{B}^{(d)^\top}(x_{1,d}) \quad \cdots \quad v_{r;I}^{(d)}\mathbf{B}^{(d)^\top}(x_{I,d})\right]\left(\begin{bmatrix}\xi_1\\ \vdots\\ \xi_I\end{bmatrix} * \begin{bmatrix}v_{r;1}^{(d)}\\ \vdots\\ v_{r;I}^{(d)}\end{bmatrix} * \begin{bmatrix}\mathbf{B}^{(\tilde{d})}(x_{i,\tilde{d}})\mathbf{z}_{\tilde{r}}^{(\tilde{d})}\\ \vdots\\ \mathbf{B}^{(\tilde{d})}(x_{i,\tilde{d}})\mathbf{z}_{\tilde{r}}^{(\tilde{d})}\end{bmatrix}\right) \\
&= \left[\mathbf{B}^{(d)^\top}(x_{1,d}) \quad \cdots \quad \mathbf{B}^{(d)^\top}(x_{I,d})\right]\left(\begin{bmatrix}v_{r;1}^{(d)}\\ \vdots\\ v_{r;I}^{(d)}\end{bmatrix} * \left(\sum_{\tilde{d}=1}^{D}\sum_{\tilde{r}=1}^{R}\begin{bmatrix}\xi_1\\ \vdots\\ \xi_I\end{bmatrix} * \begin{bmatrix}v_{r;1}^{(d)}\\ \vdots\\ v_{r;I}^{(d)}\end{bmatrix} * \begin{bmatrix}\mathbf{B}^{(\tilde{d})}(x_{i,\tilde{d}})\\ \vdots\\ \mathbf{B}^{(\tilde{d})}(x_{i,\tilde{d}})\end{bmatrix}\mathbf{z}_{\tilde{r}}^{(\tilde{d})}\right)\right) \\
&= \mathbf{A}^{(d)}\left(\left(\mathop{*}_{k=1,k\neq d}^{D}\mathbf{A}^{(k)^\top}\boldsymbol{\gamma}_r^{(k)}\right) * \boldsymbol{\xi} * \left(\sum_{\tilde{d}=1}^{D}\sum_{\tilde{r}=1}^{R}\left(\mathop{*}_{k=1,k\neq d}^{D}\mathbf{A}^{(k)^\top}\boldsymbol{\gamma}_{\tilde{r}}^{(k)}\right) * \mathbf{A}^{(d)^\top}\mathbf{z}_{\tilde{r}}^{(\tilde{d})}\right)\right).
\end{aligned}$$

By introducing the variables

$$\mathbf{Q}^{(d)} := \mathbf{A}^{(d)^\top}\boldsymbol{\Gamma}^{(d)} \tag{S1}$$

and

$$\mathbf{X}^{(d)} := \mathop{*}_{k=1,k\neq d}^{D}\mathbf{Q}^{(d)} \tag{S2}$$

we can simplify these expressions to

$$\mathbf{g}_r^{(d)} = \mathbf{A}^{(d)}\left(\mathbf{X}^{(d)}(:,r) * \boldsymbol{\eta}\right), \tag{S3}$$

$$\mathbf{w}_r^{(d)} = \mathbf{A}^{(d)}\left(\mathbf{X}^{(d)}(:,r) * \boldsymbol{\xi} * \left(\sum_{\tilde{d}=1}^{D}\sum_{\tilde{r}=1}^{R}\mathbf{X}^{(\tilde{d})}(:,\tilde{r}) * \left(\mathbf{A}^{(\tilde{d})^\top}\mathbf{z}_{\tilde{r}}^{(\tilde{d})}\right)\right)\right), \tag{S4}$$

where the parenthesis $(:,r)$ denotes the r-th column of $\mathbf{X}^{(d)}$. For the quadratic objective function in section 3, we have that

$$\boldsymbol{\eta} = \hat{\mathbf{y}} - \mathbf{y}, \qquad \boldsymbol{\xi} = \mathbf{1}_N, \tag{S5}$$

where

$$\hat{\mathbf{y}} := \begin{bmatrix} \hat{f}(\mathbf{x}_i; \boldsymbol{\Gamma}^{(1)}, \ldots, \boldsymbol{\Gamma}^{(D)}) \\ \vdots \\ \hat{f}(\mathbf{x}_i; \boldsymbol{\Gamma}^{(1)}), \ldots, \boldsymbol{\Gamma}^{(D)}) \end{bmatrix} = \left(\mathop{*}_{d=1}^{D} \mathbf{X}^{(d)} \right) \mathbf{1}_R, \qquad \text{and} \qquad \mathbf{y} := \begin{bmatrix} y_1 \\ \vdots \\ y_I \end{bmatrix}. \tag{S6}$$

In the case of the logarithmic objective function in section 4, we have the expressions

$$\boldsymbol{\eta} = \alpha \left(\sigma_\alpha \left(\hat{\mathbf{y}} \right) - \mathbf{y} \right), \qquad \text{and} \qquad \boldsymbol{\xi} = \alpha^2 \sigma_\alpha \left(\hat{\mathbf{y}} \right) * \left(1 - \sigma_\alpha \left(\hat{\mathbf{y}} \right) \right). \tag{S7}$$

1.2 Summary of computational steps

In each GN or generalized GN iteration, the gradient and it_{CG} Gramian-vector products are required, in which it_{CG} is the number of CG iterations to solve the linear systems described at the end of section 3.2 and 4.2. Let us define the shorthand notations:

$$\mathbf{H}^{(d)} := \begin{bmatrix} \mathbf{g}_1^{(d)} & \cdots & \mathbf{g}_R^{(d)} \end{bmatrix}, \qquad \mathbf{Z}^{(d)} := \begin{bmatrix} \mathbf{z}_1^{(d)} & \cdots & \mathbf{z}_R^{(d)} \end{bmatrix}, \qquad \mathbf{W}^{(d)} := \begin{bmatrix} \mathbf{w}_1^{(d)} & \cdots & \mathbf{w}_R^{(d)} \end{bmatrix}.$$

The steps for evaluating the gradient and Gramian-vector product can then be summarized as:

Step 1. Precompute $\mathbf{Q}^{(d)} \in \mathbb{R}^{I \times R}$ as defined in (S1).

Step 2. Precompute $\mathbf{X}^{(d)} \in \mathbb{R}^{I \times R}$ defined in (S2).

Step 3. Evaluate $\boldsymbol{\eta} \in \mathbb{R}^I$ and $\boldsymbol{\xi} \in \mathbb{R}^I$ according to (S5) or (S7), depending on which objective function is being considered. The vector $\hat{\mathbf{y}}$ defined in (S6) is computed as $\hat{\mathbf{y}} = \left(\mathop{*}_{d=1}^{D} \mathbf{X}^{(d)} \right) \mathbf{1}_R$.

Step 4. Evaluate $\mathbf{H}^{(d)} \in \mathbb{R}^{(M_d+1) \times R}$ and $\mathbf{W}^{(d)} \in \mathbb{R}^{(M_d+1) \times R}$ using the expressions (S3) and (S4), respectively.

Algorithm 1 provides a pseudocode on how these formulas can be evaluated in practice.

Algorithm 1 Gradient and Gramian-vector product computations.

function GRAMIANPRODUCT($\{\mathbf{Z}^{(d)}\}_{d=1}^D, \{\mathbf{A}^{(d)}\}_{d=1}^D, \{\mathbf{X}^{(d)}\}_{d=1}^D, \boldsymbol{\xi}$)
  $\mathbf{v} \leftarrow 0$
  for $d = 1 : D$ **do**
    for $r = 1 : R$ **do**
      $\mathbf{v} \leftarrow \mathbf{v} + \mathbf{X}^{(d)}[:, r] * \left({\mathbf{A}^{(d)}}^\top \mathbf{Z}^{(d)}[:, r] \right)$
  $\mathbf{v} \leftarrow \boldsymbol{\xi} * \mathbf{v}$
  $\{\mathbf{W}^{(d)}\}_{d=1}^D \leftarrow$ GRADIENT($\mathbf{v}, \{\mathbf{A}^{(d)}\}_{d=1}^D, \{\mathbf{X}^{(d)}\}_{d=1}^D$)
  return $\{\mathbf{W}^{(d)}\}_{d=1}^D$
function GRADIENT($\boldsymbol{\eta}, \{\mathbf{A}^{(d)}\}_{d=1}^D, \{\mathbf{X}^{(d)}\}_{d=1}^D$)
  for $d = 1 : D$ **do**
    for $r = 1 : R$ **do**
      $\mathbf{H}^{(d)}[:, r] \leftarrow \mathbf{A}^{(d)} \left(\mathbf{X}^{(d)}[:, r] * \boldsymbol{\eta} \right)$
  return $\{\mathbf{H}^{(d)}\}_{d=1}^D$

1.3 Complexity

As the number of iterations of the (generalized) GN algorithm is highly variable and depends, among others, on the initialization, the per-iteration complexity is derived here. Every iteration, the gradient is computed once, the function value it_{TR} times (usually once), and the Gramian-vector is computed it_{CG} times for different vectors $\mathbf{z}_r^{(d)}$ but the same variables $\mathbf{\Gamma}^{(d)}$, $d = 1, \ldots, d$, $r = 1, \ldots, R$. Typical values for it_{CG} are 15, 25, or 50.

In the derivation of the complexity, we take $N = \max_d N_d$, which typically satisfies $N \leq 4$. The number of spline basis function M_d, which grows linearly with the number of knots, is determined by $M = \max_d M_d$. The number of samples is I and the rank of the coefficient tensor is chosen to be R.

For general basis functions, precomputing $\mathbf{Q}^{(d)}$ and $\mathbf{X}^{(d)}$ in Steps 1 and 2 costs $\mathcal{O}(DIMR)$ and $\mathcal{O}\left(D^2IMR\right)$ flop, respectively. The computation of the gradient (Step 3) involves the (transformed) residual $\boldsymbol{\eta}$, which is also used in the computation of the function value, and takes $\mathcal{O}(DIR)$ flop. Finally, to compute the (generalized) GN step iteratively, the CG algorithm performs it_{CG} Gramian-vector products (Step 4), each of which takes $\mathcal{O}(DIMR)$ flop. Hence, overall the computational cost is dominated by the Gramian-vector products: $\mathcal{O}(\text{it}_{\text{CG}}DIMR)$ flop per GN iteration.

In this paper, we propose to not use general basis functions, but we use B-splines instead. This has a computational advantage as the matrices $\mathbf{A}^{(d)}$ and $\mathbf{X}^{(d)}$ are now sparse, having approximately N nonzeros per sample. By exploiting the sparsity, the complexity can effectively be reduced to $\mathcal{O}(\text{it}_{\text{CG}}DINR)$ flop per GN iteration. Hence the dependence on the potentially large number of basis functions M is removed and replaced by the low order of the B-splines N.
